# Role of LiOH in Aqueous Electrocatalytic Defluorination of Perfluorooctanoic Sulfonate: Efficient Li–F Ion Pairing Prevents Anode Fouling by Produced Fluoride

**DOI:** 10.1021/acscatal.4c04523

**Published:** 2024-10-25

**Authors:** Ziyi Meng, Madeleine K. Wilsey, Astrid M. Müller

**Affiliations:** † Materials Science Program, University of Rochester, Rochester, New York 14627, United States; ‡ Department of Chemical Engineering, University of Rochester, Rochester, New York 14627, United States; § Department of Chemistry, University of Rochester, Rochester, New York 14627, United States

**Keywords:** PFAS, PFOS, electrocatalysis, defluorination, lithium ion, ion pairing, electrode fouling, fluoride

## Abstract

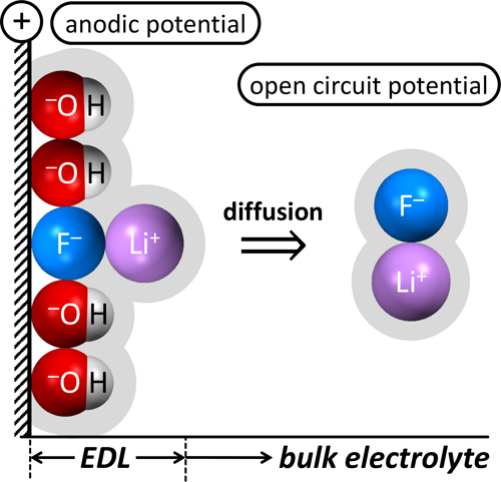

Per- and polyfluoroalkyl substances (PFAS) pose a significant environmental and health threat due to their high toxicity, widespread use, and persistence in the environment. Electrochemical methods have emerged as promising approaches for PFAS destruction, offering cost-effective and energy-efficient solutions. We established recently that electrocatalysis with nonprecious materials enabled the complete defluorination of perfluorooctanesulfonate (PFOS) in aqueous 8.0 M LiOH. Here, we reveal the mechanistic role of LiOH in the efficient aqueous electrocatalytic PFOS defluorination. Our results demonstrate that synergistic effects of high lithium and high hydroxide ion concentrations are essential for complete PFOS defluorination. Two-dimensional NMR data of electrolytes post pulsed electrolysis provide experimental evidence for Li–F ion pairing, which plays a crucial role in preventing anode fouling by produced fluoride, thus enabling sustained C–F bond cleavage. This Li–F ion pairing was increased at high pH, and elevated temperatures enhanced diffusion of Li–F ion pairs into the bulk electrolyte. High hydroxide ion concentrations additionally removed fluoride from the anode surface by competitive adsorption, corroborated by XPS data. Our findings provide quantitative mechanistic insights into the electrocatalytic defluorination process and offer a general route of enhancing the efficiency of anodic PFAS defluorination.

## 1 Introduction

Per- and polyfluoroalkyl substances (PFAS) are a class of durable synthetic chemicals widely used in consumer, commercial, and industrial products that are indispensable for decarbonization technologies.^1^ Concerns about their persistence, widespread contamination of water sources, bioaccumulation, entry into the food chain, chronic toxicity, and concomitant adverse effects on human health necessitate advanced PFAS destruction strategies.^1,2^ Efforts to mitigate the impact of PFAS contamination have been hampered by high costs and energy demands.^1^ Electrochemical approaches have emerged as promising alternatives for PFAS destruction.^1^ Photoassisted electrocatalytic defluorination offers a sustainable and cost-effective solution for PFAS degradation, by harnessing the power of radical-mediated advanced redox processes and innovative electrolyte engineering.^3^ In light of the new U.S. Environmental Protection Agency regulatory framework on PFAS drinking water standards,^4^ economically viable PFAS defluorination technologies are now more needed than ever.

We focus here on anodic PFAS defluorination. Advanced oxidation of PFAS in aqueous electrolyte requires reactive oxygen species (H_2_O_2_, HOO^•–^, ^•^OH, O^•–^, O_2_^•–^), which can be produced directly at the anode or via deep ultraviolet (UV)-light-assisted decomposition of H_2_O_2_ (or HOO^–^ at high pH). Water oxidation yields O_2_, H_2_O_2_, or ^•^OH;^5^ H_2_O_2_ oxidation produces O_2_^•–^;^6^ and O_2_ can be reduced to H_2_O_2_, ^•^OH, HOO^•–^, or O_2_^•–^ (or their deprotonated forms at high pH),^7^ which can oxidize PFAS. The cathodic aqueous reduction of dioxygen that stems from ambient air or anodic water oxidation can produce reactive oxygen species^8^ that are capable of C–F-bond cleavage, but the generation of cathodically produced reactive oxygen species through oxygen reduction is inherently limited by the solubility of oxygen in the electrolyte at ambient conditions and the oxygen reduction selectivity for hydrogen peroxide.^9^ At high applied potentials, i.e. high overpotentials, at which operation is desirable because of enhanced oxygen reduction kinetics, the 4-electron–4-proton oxygen reduction to water outcompetes the desired 2-electron–2-proton pathway to hydrogen peroxide in aqueous media, limiting the amount of reactive oxygen species that can be generated cathodically.^9^

Extensive research has been devoted to developing materials and systems for the destruction of PFAS. The chemical mineralization of perfluorocarboxylic acids is intriguing, particularly from a mechanistic perspective, but it is effective only in polar organic electrolytes, such as dimethyl sulfoxide, and it fails to degrade sulfonic acid PFAS, such as PFOS.^10^ Boron-doped diamond (BDD) is considered a benchmark material for the anodic degradation of PFOS due to its broad electrochemical window, robust chemical stability, and high oxygen evolution overpotential, all of which contribute to the rapid degradation of PFAS in water.^11^ The high cost of BDD, however, hinders the large-scale use necessary for PFAS remediation technologies.^1^ Therefore, other anode materials have emerged, including metal oxide (PbO_2_, SnO_2_, TiO_2_),^12^ mixed metal (Pd–Ru, Fe–Mn, Ce-doped Sb_2_S_3_),^13^ and functionalized carbon materials.^14^ These systems removed over 90% of PFAS but did not achieve complete defluorination, generating shorter-chain byproducts that are often more harmful than the initial PFAS.^1,15^ A common approach to increasing the efficiency of PFAS destruction is the use of additives (sulfite, sulfate, persulfate, or hydrogen peroxide) that enhance the generation of oxidants,^1,16^ albeit creating feasibility issues for scaleup.

Globally scalable, viable PFAS destruction technologies must use nonprecious materials only, operate in aqueous media with low energy consumption, and ensure complete defluorination at low concentrations without the need for additives.^1^ Our PFOS defluorination approach meets these critical criteria,^3^ representing key advancements over existing research. Utilizing nonprecious materials helps ensure that PFAS abatement technologies are practical, sustainable, and accessible.^17^ PFAS destruction in water, instead of in an organic solvent, reduces environmental risks, costs, and hazardous waste, ensuring scalable implementation.^18^ Energy efficiency is essential for lowering operational costs.^18^ Complete defluorination at low concentrations is necessary for cost-effective abatement of polluted waterways.^19^ Additives that generate auxiliary radicals to aid in PFAS destruction^1^ often require additional procurement, handling, and disposal, which can increase the overall expense of the treatment process.^20^ They may also introduce additional pollutants or byproducts that need to be managed, thereby increasing the environmental impact.^19,21^ Additive-free technologies may face fewer regulatory hurdles and receive greater acceptance from communities and stakeholders, enhancing the feasibility, effectiveness, and acceptance of PFAS destruction technologies and making them more suitable for widespread implementation.

We demonstrated the complete electrocatalytic defluorination of perfluorooctanesulfonate (PFOS) in 8.0 M LiOH, effective at PFOS concentrations as low as 27 ppm.^3^ Surfactant-free laser-synthesized [NiFe]–(OH)_2_ nanosheets on hydrophilic carbon fiber paper served as anodes, to provide high surface area and avoid mechanistic complications by surfactants.^22^ Deep UV-light-assisted defluorination was most efficient at 1.6 V vs RHE and occurred within the anode microenvironment, evident from pulsed electrolysis data, electrolyte agitation experiments, and surface composition data.^3^ X-ray photoelectron spectroscopy (XPS) data showed adsorbed C–F_2_, C–F_3_, sulfonate, O_2_, and HOO^–^, which forms the C–F bond cleaving oxidant O^•–^ with deep UV light. High concentrations of lithium and hydroxide ions were essential for effective PFOS defluorination. Our method employs only nonprecious materials in an aqueous LiOH electrolyte, making it nearly one hundred times cheaper and more energy-efficient than using BDD electrodes.^3^ This approach will aid in the remediation of contaminated environments and is poised to mitigate the long-term impact of PFAS on aquatic ecosystems, soil quality, and biodiversity. Additionally, lower-income regions around the world typically face higher levels of pollution. This makes electrocatalytic PFAS remediation ideal for decentralized implementation powered by electricity generated from solar panels, helping to restore environmental justice globally. A central novelty of our approach lies in the use of LiOH in water to enable efficient PFOS defluorination.

Here, we elucidate the mechanistic reasons why high Li^+^ and high OH^–^ concentrations are essential for efficient anodic aqueous electrocatalytic PFOS defluorination. The defluorination process produces fluoride anions that cover the positively charged anode and deactivate it if these fluoride anions are not removed from the anode surface. We provide experimental evidence by two-dimensional ^7^Li–^19^F-HOESY-NMR data that efficient Li–F ion pair formation occurred in aqueous LiOH electrolyte with high Li^+^ and high OH^–^concentrations, ergo in conditions at which PFOS defluorination was efficient. Leveraging pulsed electrolysis, effective removal of generated fluoride anions from the anode surface was facilitated by Li–F ion pair diffusion, corroborated by temperature dependence data that showed more defluorination at elevated temperature. Additionally, XPS data confirmed that high OH^–^concentrations enabled interfacial F^–^ removal by competitive adsorption. The mechanistic insights gained will enable the design of scalable processes for developing efficient, cost-effective, and widely applicable technologies for PFAS destruction.

## 2 Results and Discussion

### 2.1 Anode for PFOS Defluorination

**Figure 1:**
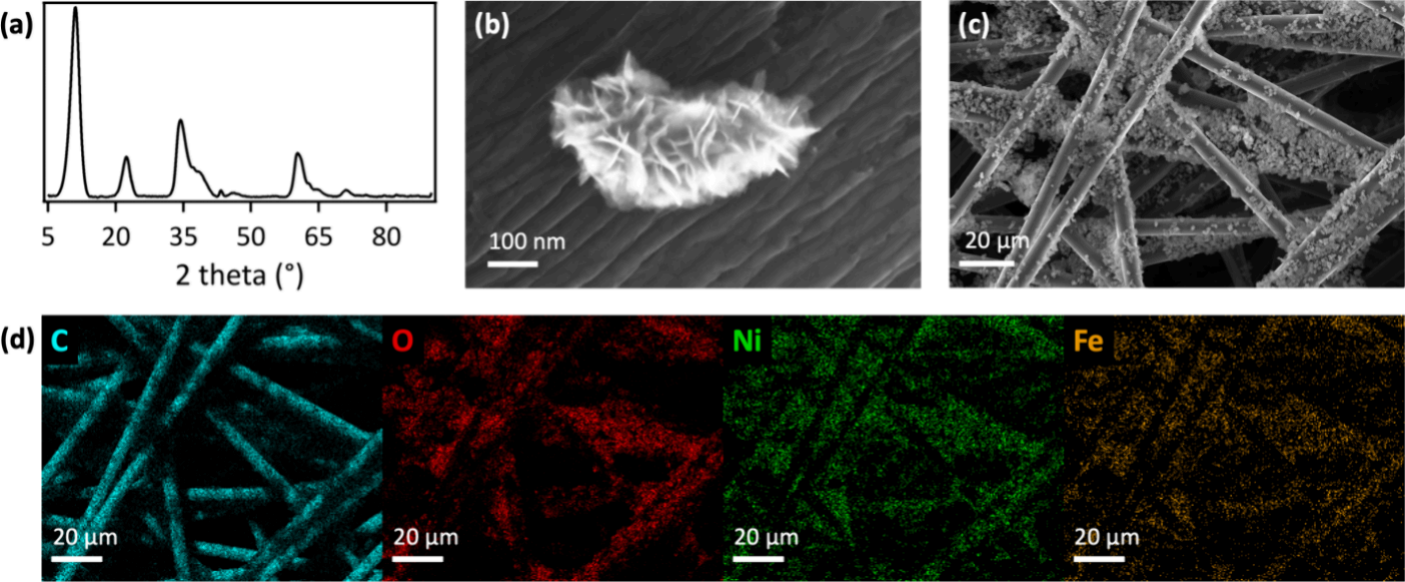
(a) XRD data of laser-made [Ni_0.75_Fe_0.25_]–(OH)_2_ nanosheets. (b, c) SEM images and (d) SEM-EDX maps of integrated anodes on hydrophilic carbon fiber paper.

Anodes for PFOS defluorination consisted of laser-made [NiFe]–(OH)_2_ nanosheets on hydrophilic carbon fiber paper. We used pulsed laser in liquid synthesis to prepare surfactant-free [NiFe]–(OH)_2_ water oxidation nanocatalysts, following a published protocol.^23,24^ The laser method does not require surfactants for size control of resulting nanoparticles, offering precise control of surface chemistries of nanocatalysts.^22^ The produced material displayed layered double hydroxide structure and nanosheet morphology, evident from X-ray diffraction (XRD) and scanning electron microscopy (SEM) imaging data (Figure 1a,b). Broadening was observed for all XRD peaks, attributable to the small crystallite size and the presence of stacking faults, including turbostratic disorder in the hydrotalcite-like structure, as previously reported for laser-synthesized [NiFe]–(OH)_2_ nanosheets.^24^ We used hydrophilic carbon fiber paper as electrode support to provide electrical contact to [NiFe]–(OH)_2_ nanosheets. The high internal surface area of 468 cm^2^ per geometric cm^2^ of carbon fiber paper^3^ enhanced PFOS defluorination, compared to a flat electrode support. Carbon fiber paper was rendered hydrophilic by oxygenation, using a rapid, green chemistry process that we developed in prior work.^25^ Briefly, our acid-free, transition-metal-free process involved sonication of carbon fiber paper in aqueous sodium dodecyl sulfate solution, followed by electrooxidation in aqueous KHCO_3_ electrolyte.^25^ Laser-made [NiFe]–(OH)_2_ nanocatalysts were electrostatically integrated with hydrophilic carbon fiber paper, resulting in an even dispersion of nanocatalysts throughout the three-dimensional structure of the carbon fibers, which effectively leveraged the high internal surface area of carbon fiber paper (Figure 1c,d). Analysis by energy-dispersive X-ray spectroscopy (EDX) showed that the laser-made [NiFe]–(OH)_2_ nanosheets exhibited a Ni:Fe ratio of 3:1. EDX spectra of laser-made [Ni_0.75_Fe_0.25_]–(OH)_2_ nanosheets on hydrophilic carbon fiber paper showed the presence of C, O, Fe, and Ni; no other elements were observed (Figure S1).

**Figure 2:**
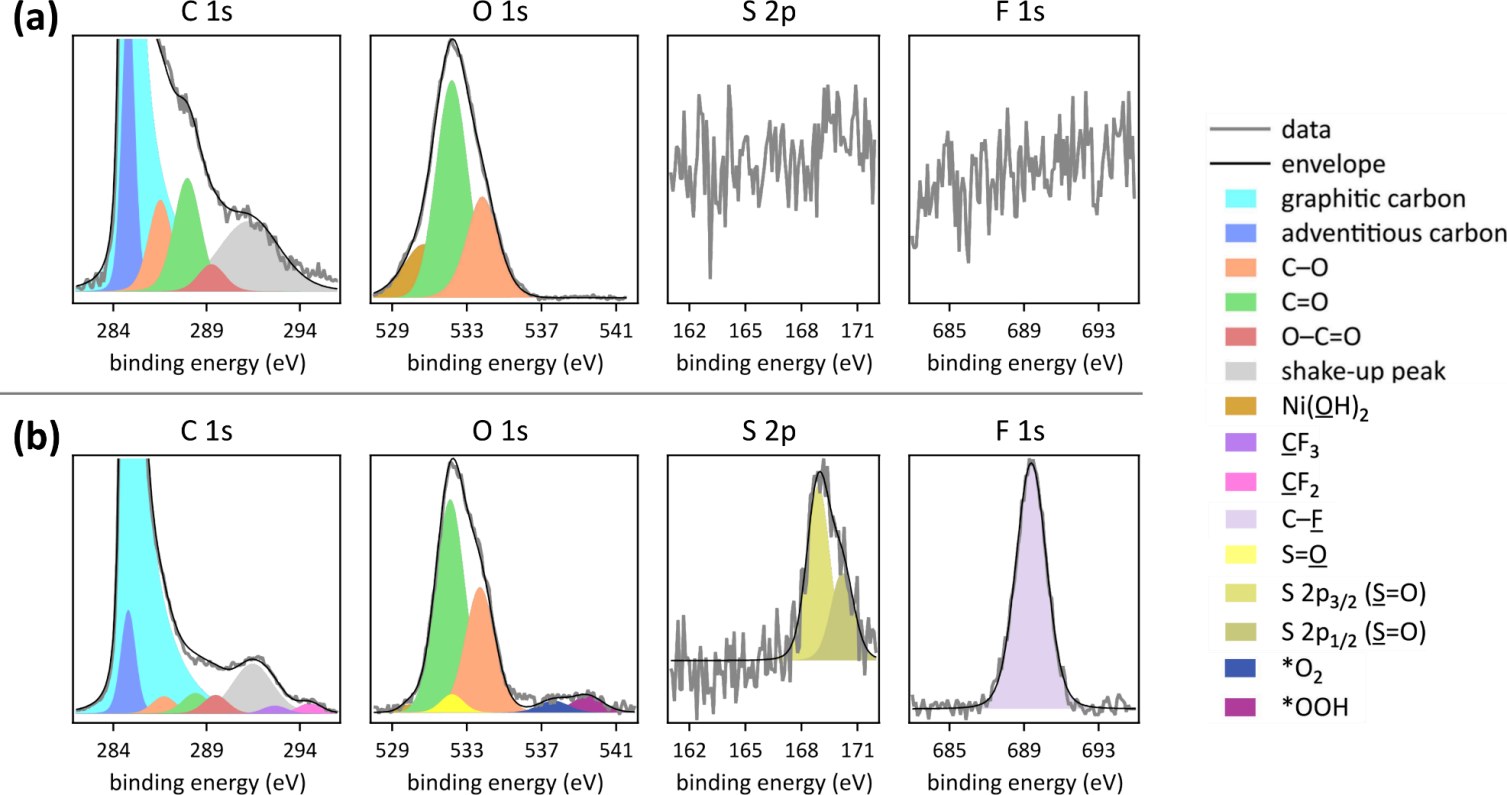
High-resolution XPS data and peak fits in the C 1s (*y*-axis magnified by a factor of 10), O 1s, S 2p, and F 1s core level regions of [Ni_0.75_Fe_0.25_]–(OH)_2_–hydrophilic carbon fiber paper anodes (a) pre electrocatalysis and (b) post pulsed electrolysis for 60 cycles (1 cycle = 1 min at 1.6 V vs RHE, followed by 5 min at open circuit potential), with deep UV light irradiation in stagnant 8.0 M aqueous LiOH electrolyte.

XPS data pre electrocatalysis and after complete PFOS defluorination in 8.0 M aqueous LiOH are shown in Figures 2 and S2. XPS data revealed the presence of the elements carbon, oxygen, nickel, and iron at anode surfaces pre and post electrocatalysis, as expected for these integrated anodes.^3^ Pre and post electrocatalysis high-resolution C 1s spectra exhibited six peaks, an asymmetrical shape, and shakeup characteristics, consistent reported graphitic carbon XPS data.^26^ The central binding energy of graphitic carbon ranged from 284.5 to 285.0 eV, consistent with reported values.^27^ Additionally, adventitious carbon was present, whose central binding energy was taken to be 284.8 eV.^28^ The remaining three peaks were attributable to carbon oxygenates, with C 1s binding energy ranges of 286.4–287.0 eV (C–O; hydroxyls, esters, and ethers), 287.4–288.0 eV (C=O; aldehydes, carbonyls, and ketones), and 288.7–289.2 eV (O–C=O; carboxyls and esters).^29,30^ Pre and post electrocatalysis O 1s data displayed two peaks with central binding energies of 531.6–532.3 eV, attributable to C=O functional groups, such as in carboxyls, aldehydes, carbonyls, esters, and ketones, and 533.0–533.7 eV, attributable to C–O species, such as in carboxyls, hydroxyls, and ethers,^29^ as observed previously.^3,25^ The carbon oxygenate C 1s peak fits were constrained to the observed carbon oxygenate O 1s atom percentages, using element-specific relative sensitivity factors resulting from photoemission cross sections and analyzer transmission of photoelectrons.^25^ Additionally, an O 1s peak at a central binding energy of 530.6 eV was present, attributable to Ni(OH)_2_ of the nanocatalyst.^31^ The corresponding Ni 2p core level region showed two Ni 2p_3/2_ and Ni 2p_1/2_ spin–orbit–split peaks with respective satellite peaks at central binding energies of 855.3 and 872.8 eV, respectively, indicative to Ni(OH)_2_.^31,32^ The Fe 2p core level regions exhibited the two Fe 2p_3/2_ and Fe 2p_1/2_ spin–orbit–split peaks at central binding energies of 711.8 and 723.1 eV, consistent with Fe(OH)_2_.^33^

XPS data after electrocatalytic defluorination of 0.5 mM PFOS in 8.0 M LiOH exhibited additional peaks in the C 1s, O 1s, F 1s, and S 2p core level regions, attributable to adsorbed C–F_2_ and C–F_3_ moieties and adsorbed sulfonate, stemming from PFOS, as well as adsorbed deprotonated hydrogen peroxide (Figure 2b).^3,34−37^ We determined atom percentages of fluorine and sulfur species relative to total surface carbon by quantifying the signals in the F 1s, and S 2p core level regions, respectively. The strong F 1s peak had a central binding energy of 689.4 eV, consistent with the reported range of C–F bonds in PFOS (688–689 eV),^34,35^ and an area that corresponded to 1.2 at % fluorine. The weak S 2p signal corresponded to 0.2 at % sulfur and showed two spin–orbit–split peaks with central binding energies of 168.8 and 170.3 eV, assignable to the S 2p_3/2_ and S 2p_1/2_ components of sulfonate S=O.^34−36^ The corresponding C 1s peaks of C–F_3_ and C–F_2_ species and O 1s peak of the sulfonate moiety appear in the same binding energy region as the stronger oxygenated carbon peaks. Nevertheless, we included these corresponding C–F_3_, C–F_2_, and sulfonate peaks to the overall C 1s and O 1s signal fits at the reported binding energies and with peak areas constrained to the atom percentages deduced from fitting the signals in the F 1s and S 2p core level regions (Figures 2b and S2). The C 1s signals of the C–F_2_ and C–F_3_ moieties have reported binding energies of 292.2 and 294.5 eV, respectively.^34^ The O 1s peak of the PFOS sulfonate S=O moieties appears at a central binding energy of 532.2 eV.^37^ The deprotonated hydrogen peroxide, observed in the O 1s core level region as a peak with a central binding energy of 539.5 eV, consistent with prior work,^3^ originated from the two-proton–two-electron alkaline water oxidation to HOO^–^, which is energetically accessible at the conditions used here.^3^ We established previously that this deprotonated hydrogen peroxide together with deep UV light irradiation generated the oxidant O^•–^, which defluorinated PFOS within the anode microenvironment.^3^ After complete PFOS defluorination in 8.0 M LiOH, no Li 1s signal was detectable (Figure S2).

### 2.2 Dependence of PFOS Defluorination on the Identity and Concentration of Ions

We conducted pulsed electrocatalysis experiments with [Ni_0.75_Fe_0.25_]–(OH)_2_–hydrophilic carbon fiber paper anodes and 0.5 mM PFOS in stagnant aqueous electrolyte at 1.6 V vs RHE, with deep UV light irradiation. Pulsed electrolysis consisted of 60 cycles, in which one cycle was comprised of a 1 min interval at +1.6 V vs RHE, followed by a 5 min interval at open circuit potential. We used aqueous 6.0 M [LiOH]_*x*_–[LiClO_4_]_(1–*x*)_ or 6.0 or 8.0 M [LiOH]_*x*_–[NaOH]_(1–*x*)_ (*x* = 1, 0.25, 0.5, 0.75, 0) electrolytes, to disentangle the effect of high Li^+^ and high OH^–^ concentrations in the electrolyte on PFOS defluorination. The [LiOH]_*x*_–[LiClO_4_]_(1–*x*)_ electrolytes kept the Li^+^ concentration constant, while varying the OH^–^ concentration, and spanned a pH range of 7.8 (pure LiClO_4_) to 14.8 (pure LiOH). The solubility limit of LiClO_4_ in water at standard conditions dictated use of 6.0 M solutions. The [LiOH]_*x*_–[NaOH]_(1–*x*)_ electrolytes kept the OH^–^ concentration constant, while varying the Li^+^ concentration, and had pH values of 14.8 (6.0 M [LiOH]_*x*_–[NaOH]_(1–*x*)_) or 14.9 (8.0 M [LiOH]_*x*_–[NaOH]_(1–*x*)_). We quantified fluoride concentrations using a fluoride ion selective electrode^38^ combined with a total ionic strength adjustment buffer (TISAB) that sufficiently acidified the solution to prevent hydroxide ion interference,^3^ in accordance with the recommendations of the manufacturer of the fluoride ion selective electrode.^39^ This solution acidification by the TISAB additionally cleaved any (Li or Na)–F ion pairs that were stable in strongly alkaline electrolytes, to release all electrocatalytically generated fluoride ions into the analyte.

**Figure 3:**
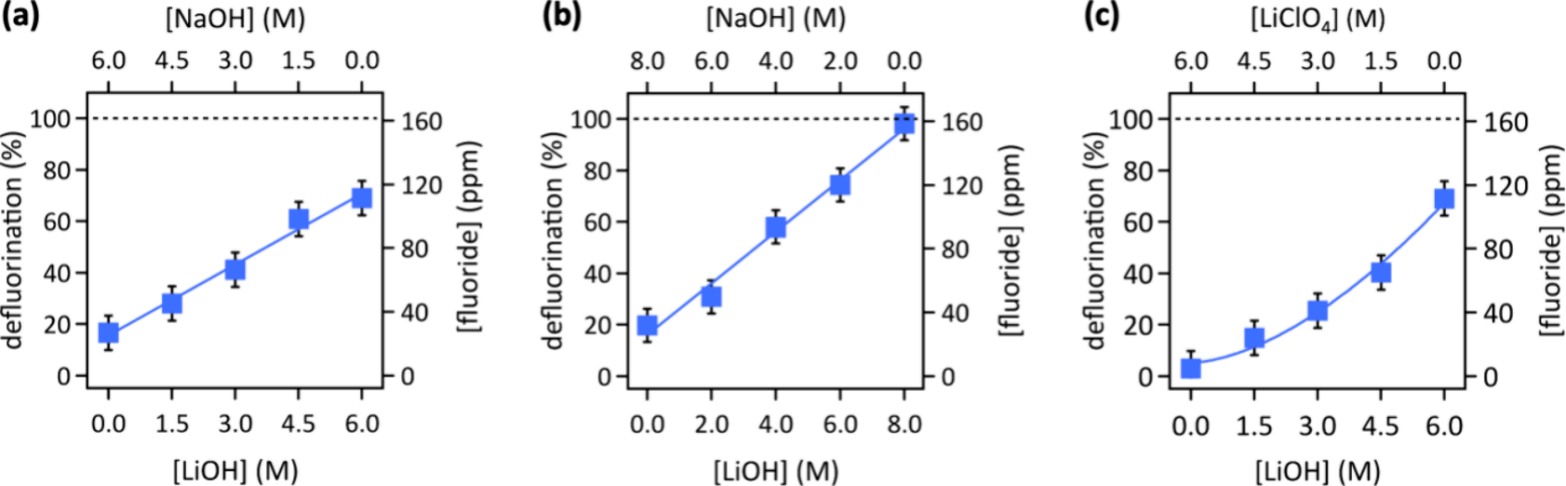
PFOS defluorination in (a) 6.0 M or (b) 8.0 M aqueous [LiOH]_*x*_–[NaOH]_(1–*x*)_ or (c) 6.0 M aqueous [LiOH]_*x*_–[LiClO_4_]_(1–*x*)_ (*x* = 1, 0.25, 0.5, 0.75, 0) electrolytes. Conditions: pulsed electrolysis (60 cycles, with 1 cycle = 1 min at 1.6 V vs RHE, followed by 5 min at open circuit potential, deep UV light irradiation) in stagnant electrolyte. The lines in (a) and (b) are linear fits, the line in (c) is a power law fit. Error bars represent standard deviations of triplicate measurements.

When the Li^+^ concentration was varied at constant OH^–^ concentration, we found that the PFOS defluorination was most efficient at highest Li^+^ concentration in the 6.0 or 8.0 M aqueous [LiOH]_*x*_–[NaOH]_(1–*x*)_ (*x* = 1, 0.25, 0.5, 0.75, 0) electrolytes. The defluorination exhibited a linear dependence on the Li^+^ concentration (Figure 3a,b). Defluorination was higher in the 8.0 M than 6.0 M aqueous [LiOH]_*x*_–[NaOH]_(1–*x*)_ electrolyte. For varying the OH^–^ concentration at constant Li^+^ concentration, we found that the PFOS defluorination was most efficient at highest OH^–^ concentration in the 6.0 M aqueous [LiOH]_*x*_–[LiClO_4_]_(1–*x*)_ (*x* = 1, 0.25, 0.5, 0.75, 0) electrolytes (Figure 3c).

Rate analysis of log–log plots of the data of Figure 3 showed a first order dependence of fluoride production on the OH^–^ concentration (slope: 1.14 ± 0.11), and less than first order dependence of obtained fluoride concentration on the Li^+^ concentration (Figure S3). Interestingly, the slope of F^–^vs Li^+^ concentration data was larger in 8.0 M than in 6.0 M aqueous [LiOH]_*x*_–[NaOH]_(1–*x*)_ electrolyte (0.82 ± 0.04 vs 0.67 ± 0.06), suggesting a cooperative effect of OH^–^ and Li^+^ ions.

### 2.3 Evidence for Li–F Ion Pairing

We collected two-dimensional (^7^Li or ^23^Na)–^19^F-HOESY-NMR^40^ data of post electrocatalysis bulk electrolytes to quantify (Li or Na)–F ion pairing as a function of the Li^+^ or OH^–^ ion concentration. Correlation of these 2D-NMR data to the observed PFOS defluorination allowed us to gain a quantitative mechanistic understanding of the role of Li–F pairing in PFOS defluorination efficiency.

**Figure 4:**
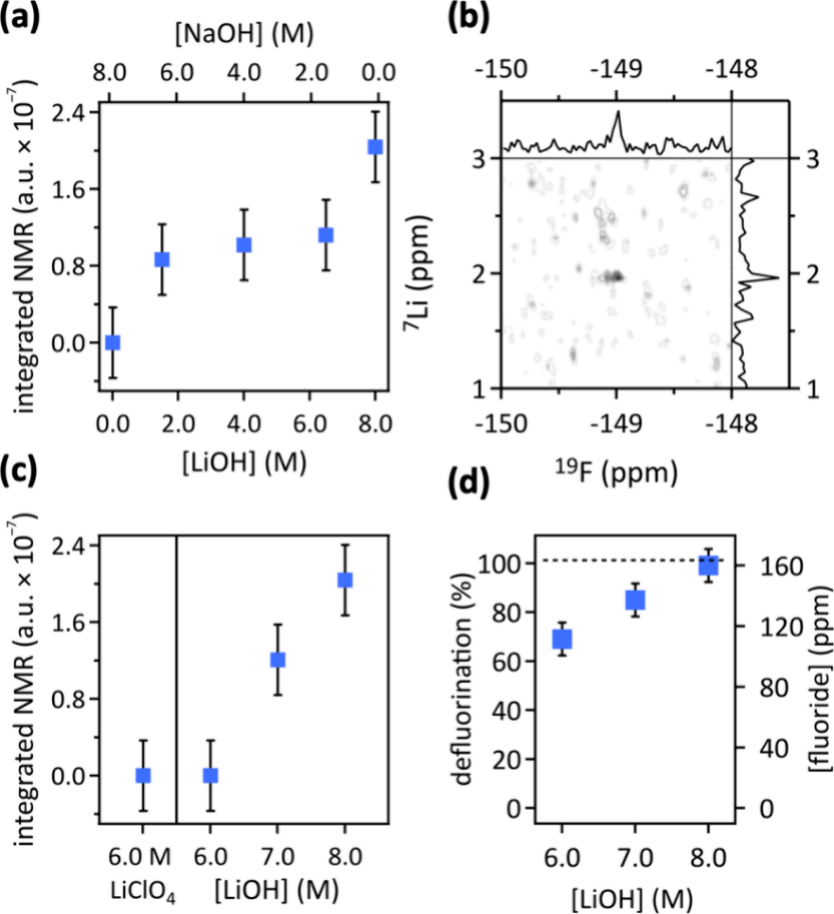
(a, c) Integrated 2D-^7^Li–^19^F-HOESY-NMR cross peak volumes as a function of the electrolyte. (b) 2D-^7^Li–^19^F-HOESY-NMR data of PFOS defluorination in 8.0 M aqueous LiOH (see Figure S4 for more 2D-NMR data). Error bars of NMR integrations are standard deviations of volume fits. (d) PFOS defluorination as a function of the LiOH concentration. Conditions: pulsed electrolysis (60 cycles, with 1 cycle = 1 min at 1.6 V vs RHE, followed by 5 min at open circuit potential, deep UV light irradiation) in stagnant electrolyte. Error bars represent standard deviations of triplicate measurements.

In [LiOH]_*x*_–[NaOH]_(1–*x*)_ (*x* = 1, 0.25, 0.5, 0.75, 0) electrolytes with *x* ≥ 0.25, we observed ^7^Li–^19^F cross peaks in ^7^Li–^19^F-HOESY-NMR data (Figures 4b and S4), providing evidence for Li–F ion pairing in electrolytes with 8.0 M OH^–^ and at least 2.0 M Li^+^. Integration of the volumes of observed cross peaks allowed us to quantify Li–F ion pairing as a function of the chemical identity and concentration of ions in the electrolyte (Figure 4a,c). In [LiOH]_*x*_–[NaOH]_(1–*x*)_ (*x* = 1, 0.25, 0.5, 0.75, 0) electrolytes, increasing the Li^+^ concentration correlated with increasing signal strength of the ^7^Li–^19^F cross peak in a sigmoidal fashion (Figure 4a). At a constant OH^–^ concentration of 8.0 M and Li^+^ concentrations increasing from 2.0 to 6.0 M, we observed a 1.3× increase of the signal strength of the ^7^Li–^19^F cross peak. In contrast, increasing the Li^+^ concentration from 6.0 to 8.0 M enhanced the signal strength of the ^7^Li–^19^F cross peak 2.1×, corroborating the cooperative effect of OH^–^ and Li^+^ ions observed in the PFOS defluorination data (Figure 3). Comparable enthalpies of hydration for Li^+^ and F^–^ of −520 and −505 kJ mol^–1^, respectively,^41^ facilitate the formation of Li–F ion pairs^42,43^ in aqueous electrolytes containing Li^+^.^44^ An increase in ion pairing enthalpies has been reported at elevated pH levels,^45^ aligning with our observation of a synergistic impact of OH^–^ and Li^+^ ions on PFOS defluorination (Figure S3). In aqueous LiOH electrolytes with concentrations that increased from 6.0 to 8.0 M, we observed a linear increase of the signal strength of the ^7^Li–^19^F cross peak and a linear increase in PFOS defluorination from (69 ± 6.7) to (99 ± 6.7) % (Figure 4c,d). The ^7^Li–^19^F cross peak showed 1.7× more signal at 8.0 M than at 7.0 M LiOH (Figure 4c). In contrast, in aqueous 8.0 M NaOH, we did not observe a cross peak in ^23^Na–^19^F-HOESY-NMR data (Figure S4), because Li–F ion pairing is stronger than Na–F ion pairing.^43^

In both 6.0 M LiOH or LiClO_4_ electrolyte, we did not observe ^7^Li–^19^F cross peaks in 2D-NMR data, but defluorination was 22.3× higher in 6.0 M LiOH than in 6.0 M LiClO_4_ electrolyte. This suggests that a different mechanism than Li–F ion pairing contributed to efficient defluorination at high OH^–^ concentration, namely competitive adsorption of OH^–^ ions at the [Ni_0.75_Fe_0.25_]–(OH)_2_–hydrophilic carbon fiber paper anode. Competition between aqueous OH^–^ and F^–^ ions for adsorbent sites has been reported on uncharged oxygenated graphitic carbon, with negligible F^–^ adsorption at high pH.^46^ At 6.0 M OH^–^ concentration, this competitive adsorption of OH^–^ ions at the anode apparently inhibited adsorption of generated F^–^ at the anode surface, thus limiting electrode fouling as PFOS defluorination proceeded. In contrast, the OH^–^ concentration in 6.0 M pH 7.8 LiClO_4_ electrolyte was 6.3 × 10^–7^ M, allowing detrimental anodic F^–^ adsorption. Halides inhibit perchlorate adsorption on activated carbon.^47^ As a result, ClO_4_^–^ ions are unable to completely displace the F^–^ ions from the anode surface, aided by the larger anion volume of ClO_4_^–^ of 0.082 nm^3^, compared to that of F^–^ of 0.025 nm^3^.^48^

**Figure 5:**
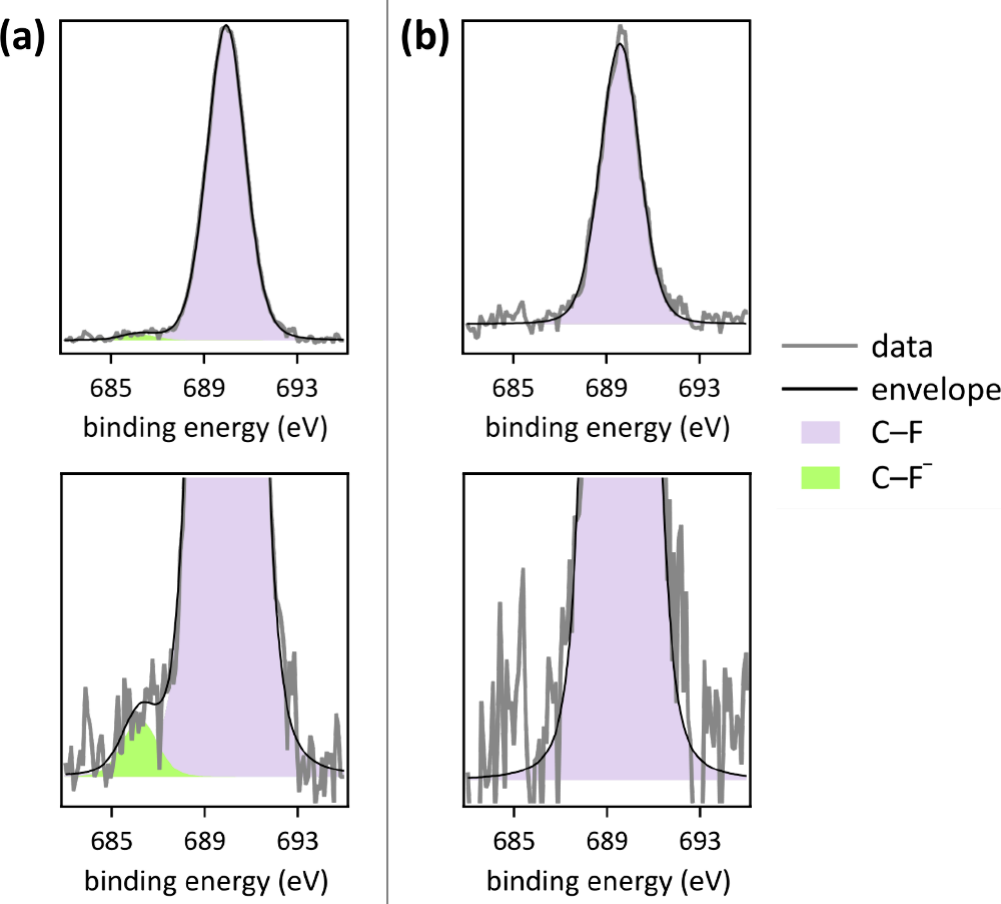
High-resolution XPS data and peak fits in the F 1s core level region (bottom: *y*-axis magnified by a factor of 10) of [Ni_0.75_Fe_0.25_]–(OH)_2_–hydrophilic carbon fiber paper anodes post pulsed electrolysis (60 cycles, with 1 cycle = 1 min at 1.6 V vs RHE, followed by 5 min at open circuit potential, deep UV light irradiation) in stagnant 6.0 M aqueous (a) LiClO_4_ and (b) LiOH electrolyte.

XPS data of anodes after PFOS defluorination electrocatalysis in 6.0 M LiClO_4_ electrolyte provide evidence for the presence of adsorbed F^–^ ions, whereas fluoride was absent at anodes after electrocatalysis in 6.0 M LiOH electrolyte (Figure 5). This supports our mechanistic proposal that competitive adsorption of OH^–^ ions at the [Ni_0.75_Fe_0.25_]–(OH)_2_–hydrophilic carbon fiber paper anode at high pH assisted PFOS defluorination by removal of produced fluoride from the anode. After PFOS defluorination electrocatalysis in 6.0 M LiClO_4_ electrolyte, the F 1s core level region displayed a peak with a central binding energy of 686.1 eV, attributable to adsorbed fluoride,^49,50^ in addition to the strong peak with a central binding energy of 689.4 eV, assignable to C–F bonds of PFOS^34,35^ (Figure 5a). In contrast, we did not detect adsorbed fluoride in F 1s XPS data after PFOS defluorination electrocatalysis in 6.0 or 8.0 M LiOH electrolyte (Figures 5b, S2 and S5). The peak assigned to adsorbed fluoride (green peak in Figure 5a) amounted to 0.07 at % F^–^ relative to total surface carbon. We included a corresponding adsorbed fluoride peak into the overall C 1s peak fitting of XPS data post electrocatalysis in 6.0 M LiClO_4_ electrolyte, with an area constrained to this atom percentage and a central binding energy of 288.4 eV^50^ (Figure S6). Additionally, after PFOS defluorination electrocatalysis in 6.0 M LiClO_4_ electrolyte, two spin–orbit–split peaks were observed in the Cl 2p core level region, with central binding energies of 208.7 and 210.4 eV, attributable to the Cl 2p_3/2_ and Cl 2p_1/2_ components of adsorbed perchlorate.^51^ We included the presence of ClO_4_^–^ into the overall fit of the O 1s core level region as a peak with a central binding energy of 533.6 eV (Figure S6).^52,53^

PFOS defluorination at [Ni_0.75_Fe_0.25_]–(OH)_2_–hydrophilic carbon fiber paper anodes was (69 ± 6.7) or (3.1 ± 6.7) % efficient in 6.0 M LiOH or LiClO_4_ electrolyte, respectively. In both cases, a Li 1s XPS signal was detectable (Figures S5 and S6). The Li 1s peak had a central binding energy of 56.9 eV, assignable to Li^+^.^53^ Peak area quantification revealed that 2.5 or 16.4 at % Li^+^ relative to total surface carbon was present after PFOS defluorination in 6.0 M LiOH or LiClO_4_ electrolytes, respectively. After complete PFOS defluorination in 8.0 M LiOH electrolyte, an analog Li 1s signal was absent (Figure S2). This suggests that high bulk electrolyte OH^–^ concentrations effectively kept Li^+^ and F^–^ ions from the anode surface, likely by specific adsorption of OH^–^ ions at the anode. While much is known about lithium ion intercalation into graphite in the context of lithium ion batteries with nonaqueous electrolytes,^54^ reports for aqueous systems are scarce and limited to halogen intercalation.^55^ Aqueous fluoride can intercalate into graphite.^56^ Here, fluoride, together with surface adsorbed perchlorate, as observed after PFOS defluorination in 6.0 M aqueous LiClO_4_ electrolyte, apparently attracted Li^+^ to the anode surface, possibly for charge balance reasons.

### 2.4 Temperature Dependence of PFOS Defluorination

**Figure 6:**
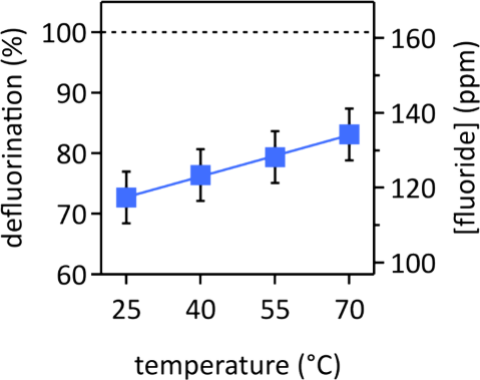
PFOS defluorination in 8.0 M aqueous LiOH as a function of the electrolyte temperature. Conditions: pulsed electrolysis (10 cycles, with 1 cycle = 1 min at 1.6 V vs RHE, followed by 5 min at open circuit potential, deep UV light irradiation) in stagnant electrolyte. Line is a linear fit. Error bars represent standard deviations of triplicate measurements.

In addition to these room-temperature experiments, we obtained PFOS defluorination data in 8.0 M aqueous LiOH electrolyte at three elevated temperatures, to elucidate the role of Li–F ion pair diffusion on the defluorination efficiency. For these temperature dependence experiments, we used only 10 instead of 60 pulsed electrolysis cycles, because we did not want to completely defluorinate PFOS at room temperature. We found that the PFOS defluorination efficiency increased linearly as a function of electrolyte temperature between 25 and 70 °C (Figure 6). We chose this temperature range such that the highest temperature was significantly below the boiling point of the electrolyte at standard conditions, which we measured to be (108 ± 1) °C, to prevent bubble formation. Bubbles would have given rise to electrolyte agitation, which would have added convection as a mass transport mechanism, thereby decreasing the thickness of the electrochemical double layer^57^ and complicating the overall analysis of the electrocatalytic process. We found in prior work that a thick electrochemical double layer, achieved by leaving the electrolyte stagnant, was required for efficient PFOS defluorination in 8.0 M aqueous LiOH electrolyte.^3^

### 2.5 Mechanistic Understanding of Electrocatalytic Aqueous LiOH Promoted Defluorination of PFOS

**Figure 7:**
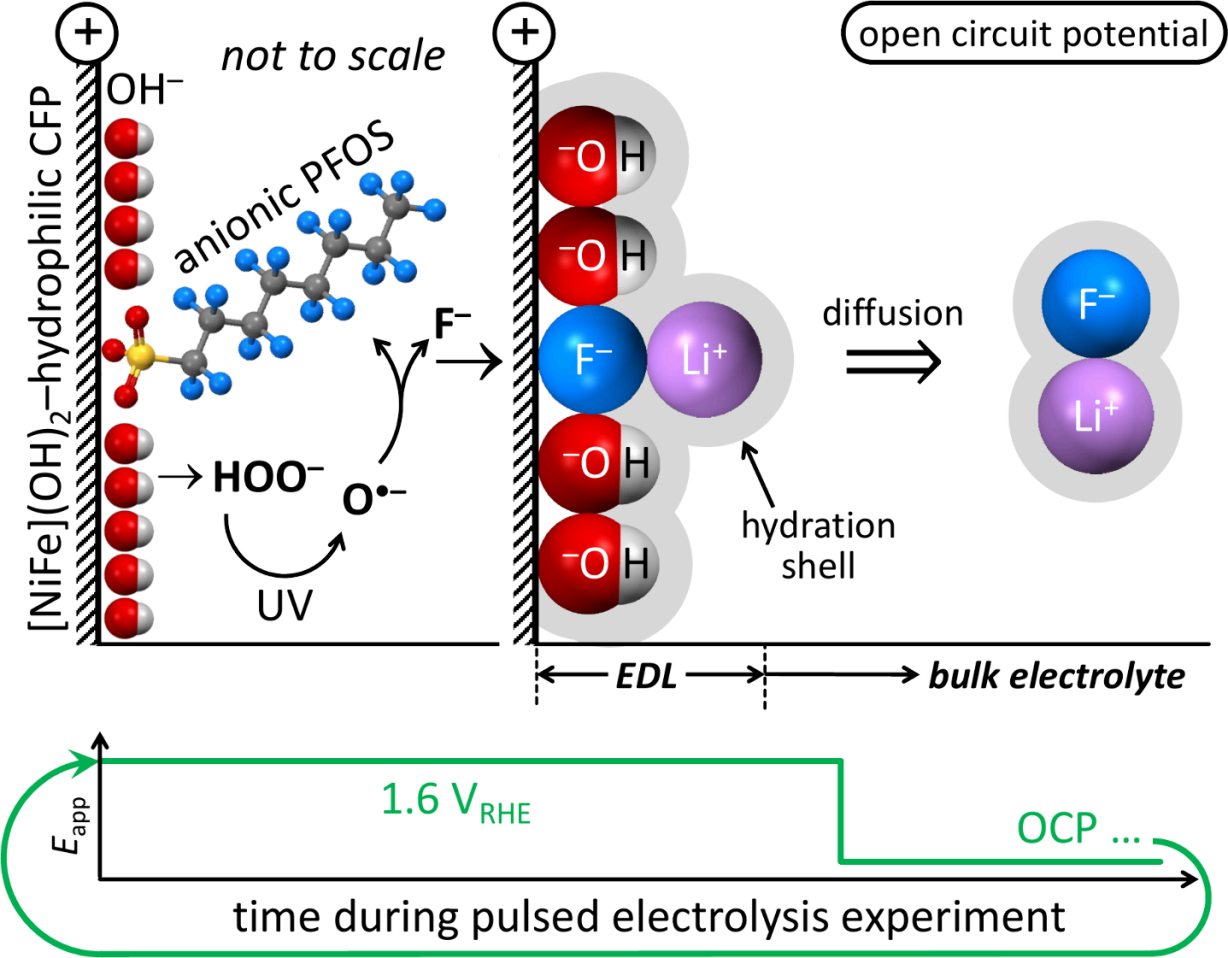
Schematic of how Li–F ion pairing, combined with pulsed electrolysis and competitive anodic adsorption of OH^–^ ions, facilitates the removal of fluoride from the anode surface. CFP, carbon fiber paper; *E*_app_, applied potential; EDL, electrochemical double layer; OCP, open circuit potential. Element colors: H (white), Li (purple), C (gray), O (red), F (blue), S (yellow).

Our data provide a quantitative mechanistic understanding of why high Li^+^ and high OH^–^ concentrations are key for complete PFOS defluorination by electrocatalysis at laser-made [Ni_0.75_Fe_0.25_]–(OH)_2_–hydrophilic carbon fiber paper anodes in aqueous LiOH electrolyte. PFOS is anionic at the pH values used here (≥7.8) because the p*K*_a_ value of PFOS is −3.27.^1^ PFOS defluorination occurs in the anode microenvironment through an advanced oxidation process by the oxidant O^•–^ that was electrocatalytically regenerated within the anode microenvironment by alkaline water oxidation and deep UV light irradiation.^3^ The cleavage of PFOS C–F bonds releases F^–^ ions into the anode microenvironment,^3^ and those F^–^ ions can specifically adsorb at the anode, thereby blocking surface sites that are needed as adsorbent sites for the adsorption of unreacted PFOS anions and as active sites for water oxidation electrocatalysis. This specific adsorption of produced F^–^ ions results in electrode fouling, which fundamentally limits PFOS defluorination. Yet, we observed complete PFOS defluorination by quantification of F^–^ in the bulk electrolyte,^3^ suggesting that an effective mechanism existed for F^–^ desorption from the anode surface and F^–^ diffusion from the electrochemical double layer into the bulk electrolyte. The F^–^ ion desorption and diffusion was efficient even though we kept the electrolyte stagnant because we found previously that a thick electrochemical double layer was beneficial for complete PFOS defluorination.^3^ Our results show that this F^–^ removal from the anode occurred by Li–F ion pairing at high pH (Figures 4 and S4), combined with competitive anodic adsorption of OH^–^ ions, and diffusion of these Li–F ion pairs into the bulk electrolyte (Figure 6), which prevented the readsorption of the negatively charged F^–^ ions at the positively charged anode. Leveraging pulsed electrolysis enabled F^–^ removal from the anode during the time intervals at open circuit potential and the adsorption of unreacted PFOS anions during the next anodic potential interval. This way, C–F bond cleavage was sustained, without electrode fouling by produced fluoride (Figure 7).

The schematic in Figure 7 illustrates the complex electrode processes during PFOS defluorination in aqueous LiOH electrolyte. Alkaline water oxidation at laser-made [Ni_0.75_Fe_0.25_]–(OH)_2_–hydrophilic carbon fiber paper anodes, assisted by deep UV light irradiation, enables the production of O^•–^ oxidants,^3^ which cleave PFOS C–F bonds, releasing fluoride. These F^–^ ions compete with OH^–^ ions for specific adsorption sites at the anode surface. This layer of specifically adsorbed anions attracts lithium cations from the electrolyte. Similar hydration enthalpies^41^ of Li^+^ and F^–^ enable Li–F ion pairing^42,43^ in Li^+^-containing aqueous electrolytes.^44^ Enhancement of ion pairing enthalpies occurs at higher pH,^45^ consistent with our finding of a cooperative effect of OH^–^ and Li^+^ ions in PFOS defluorination (Figures 3 and S3). Additionally, the high pH value, and concomitant high OH^–^ ion concentration, facilitates F^–^ desorption from the anode, which has been attributed to the weakened electrostatic forces between the sorbent surface and F^–^ at high pH.^58^ Our observation of adsorbed fluoride in XPS data after PFOS defluorination only in 6.0 M LiClO_4_ but not in 6.0 or 8.0 M LiOH corroborates that the high OH^–^ ion concentrations of the LiOH electrolytes effectively displace fluoride from the anode–electrolyte interface. The Li–F ion pairing creates charge-neutral fluoride species, enabling fluoride desorption from the positively charged anode surface, and the Li–F ion pairs diffuse into the bulk electrolyte, effectively preventing anode fouling by produced F^–^ ions. Additionally, pulsed electrolysis facilitated the Li–F ion pairing because switching the electrode from the anodic potential to open circuit potential removed the positive charges at the anode interface with the electrolyte, eliminating the electric-field-dependent electrostatic attraction of F^–^ anions to the electrode, thus weakening the affinity between fluoride and the electrode, which in turn enables Li–F ion pairing.

In pulsed electrolysis, a constant potential (here 1.6 V vs RHE) is applied briefly (sec–min; ON time), followed by an interval at open circuit potential for minutes (OFF time),^59^ to allow the electrochemical double layer to re-establish equilibrium;^60^ this ON–OFF time cycle is repeated several times. Pulsed electrolysis (1 min ON time, 5 min OFF time) was crucial for efficient defluorination of PFOS in aqueous LiOH.^3^ The electrolyte composition governs the structure of the electrochemical double layer; hydrated Li^+^ has a larger Stokes radius than hydrated Na^+^.^61^ Smaller hydrated cations enhance accumulation of cations in the outer Helmholtz plane of the electrochemical double layer, increasing the interfacial electric field,^61^ but the hydration enthalpies^41^ of Li^+^, Na^+^, and F^–^ energetically favor Li–F over Na–F ion pairing.^42,43^

Our observation that the PFOS defluorination efficiency increased linearly as a function of electrolyte temperature (Figure 6) corroborates that diffusion, particularly that of Li–F ion pairs, played a major role in the defluorination mechanism by transporting produced fluoride away from the anode surface. The molecular diffusion flux is proportional to the temperature of the liquid.^62^ Without any agitation or stirring, as was the case here, diffusion is the dominant mass transport process for charge-neutral species, such as the Li–F ion pairs of this work, both at anodic potential and at open circuit potential. In contrast, mass transport of ions occurs by diffusion only at open circuit potential, whereas a combination of diffusion and drift operate at applied potential.^57^

## 3 Conclusions

We prepared laser-made [Ni_0.75_Fe_0.25_]–(OH)_2_ nanosheets immobilized on hydrophilic carbon fiber paper as anodes for aqueous PFOS defluorination. Complete photoassisted electrocatalytic PFOS defluorination was achieved in aqueous 8.0 M LiOH electrolyte. Systematic variation of the Li^+^ concentration at constant OH^–^ concentration, together with variation of the OH^–^ concentration at constant Li^+^ concentration, allowed us to disentangle the roles of high Li^+^ and high OH^–^ concentrations on PFOS defluorination. We found that synergistic effects of high Li^+^ and high OH^–^ concentrations are essential for efficient PFOS defluorination.

Mechanistically, PFOS defluorination occurred in the anode microenvironment through an advanced oxidation process by the oxidant O^•–^ that was electrocatalytically regenerated within the anode microenvironment by alkaline water oxidation and deep UV light irradiation.^3^ This process cleaves the C–F bonds of PFOS, releasing fluoride into the anode microenvironment. The produced F^–^ ions can specifically adsorb at the anode and foul it, preventing subsequent adsorption of unreacted PFOS anions and blocking active sites for water oxidation electrocatalysis. Our results establish the mechanisms by which these generated F^–^ ions are removed from the anode surface.

Two-dimensional ^7^Li–^19^F-HOESY-NMR data demonstrate that efficient Li–F ion pair formation occurred in aqueous LiOH electrolyte with high Li^+^ and high OH^–^ concentrations, ergo in conditions at which PFOS defluorination was complete. Cooperative effects of Li^+^ and OH^–^ ions enhanced Li–F ion pairing. High Li^+^ concentrations increased Li–F ion pairing. At high OH^–^ concentrations, Li–F ion pairing was further increased, supporting our observation of a synergistic impact of OH^–^ and Li^+^ ions on PFOS defluorination. PFOS defluorination increased linearly as a function of temperature, indicating that diffusion played a role in PFOS defluorination. Pulsed electrolysis facilitated Li–F ion pair diffusion away from the anode, by switching the electrode from the anodic potential to open circuit potential to disrupt the electrostatic attraction between F^–^ anions and the electrode, enabling Li–F ion pairing in the anode microenvironment. Simultaneously, at high OH^–^ concentrations, and thus high pH, OH^–^ ions competitively adsorbed at the anode and displaced fluoride from the anode surface, evident from enhanced PFOS defluorination in neat aqueous LiOH, compared to mixed LiClO_4_ and LiOH electrolytes. XPS data confirmed the inability of perchlorate ions to completely displace fluoride ions at the anode interface, verified by the detection of adsorbed fluoride in XPS data after electrocatalysis in neat (pH 7.8) 6.0 M LiClO_4_ electrolyte, whereas fluoride was absent after electrocatalysis in high pH electrolytes.

Overall, our findings establish the mechanisms underlying the superior performance of aqueous LiOH electrolyte in PFOS defluorination, highlighting the complex interplay of Li^+^, F^–^, and OH^–^ ions, and paving the way toward more efficient and sustainable PFAS defluorination technologies.

## 4 Materials and Methods

All chemicals were used as received. Deionized water with a resistivity of ≥17.5 MΩ · cm was sourced from a Thermo Scientific Barnstead Smart2Pure Pro UV/UF 15 LPH Water Purification System. Experiments were conducted at room temperature and in ambient air. Glassware was cleaned with aqua regia, thoroughly rinsed, and dried before use. Error bars represent standard deviations of triplicate measurements. Data analysis and graphing were carried out using Igor Pro 8.04 (Wavemetrics), unless specified otherwise.

### 4.1 Anode Preparation

We used laser-made [NiFe]–(OH)_2_ nanosheets immobilized on hydrophilic carbon fiber paper as anodes. The process to prepare hydrophilic carbon fiber paper is described elsewhere.^25^ Briefly, the process modified the surfaces of carbon fiber paper, purchased from FuelCellStore (AvCarb MGL190, with 78% porosity), via sonication in a 1.0 M aqueous solution of sodium dodecyl sulfate, followed by electrooxidation in a 0.1 M aqueous KHCO_3_ electrolyte at pH 8.7 and +1.63 V vs Ag/AgCl for a duration of 20 min.^25^ The electrodes used had dimensions of 3.0 cm (length) by 3.0 cm (width), resulting in a geometric electrode area of 9.0 cm^2^ and a carbon surface area of 4200 cm^2^.^3^

Laser-made [NiFe]–(OH)_2_ nanosheets were obtained following a protocol reported elsewhere.^23^ In short, we used 90 mJ, 8 ns pulses at 355 nm, produced by the third harmonic of a 10 Hz Q-switched Nd:YAG laser (Spectra-Physics Quanta-Ray LAB-190), to irradiate a suspension of iron powder (Alfa, −200 mesh, 99+%) in a 10 mL solution of 3.0 M nickel nitrate (Alfa, 98%) in water in a 30 mL glass beaker at room temperature in ambient air for a duration of 60 min. After the laser synthesis, any unreacted iron powder was separated from the aqueous suspension of produced [NiFe]–(OH)_2_ nanoparticles using a strong magnet. The solid nanoparticulate powder was isolated through centrifugation, washed first five times with water, and then twice with acetone (VWR). The nanopowder was then dried under house vacuum. Following this, suspensions of the nanoparticulate powder at a concentration of 2 mg mL^–1^ in water were vortexed, drop-cast onto hydrophilic carbon fiber supporting electrodes, and dried under a heat lamp at 60 °C in ambient air.

### 4.2 Electrocatalysis

Details of the electrocatalysis setup are reported elsewhere.^3^ Briefly, electrochemical tests were conducted in ambient air at room temperature using a standard Pyrex three-electrode, 50 mL electrochemical cell with a Teflon lid, which remained above the liquid surface, and a flat quartz window for UV light exposure (254 nm, 40 mW cm^–2^). The anodes were laser-made [NiFe]–(OH)_2_ nanosheets immobilized on hydrophilic carbon fiber paper (444 μg cm^–2^_geo_ catalyst mass loading), and the cathodes were neat hydrophilic carbon fiber paper. A Pt wire pseudoreference electrode, calibrated in each electrolyte against a hydrogen reference electrode (Gaskatel HydroFlex), was used. Platinum wire has been shown to be a suitable and stable reference electrode in various electrochemical systems.^63^ The anode and cathode were positioned 16 mm apart and centered in the cell, with electrical connections made using aluminum clips and a potentiostat. Data are reported relative to the reversible hydrogen electrode (RHE), without correction for uncompensated resistance losses. Chronoamperometry data were collected at an anodic applied potential of 1.6 V vs RHE. In pulsed electrolysis, one cycle denotes one ON time of 1 min at an applied potential of 1.6 V vs RHE followed by one OFF time of 5 min at open circuit potential. The cell was filled with 40 mL electrolyte.

Aqueous electrolytes were prepared by adding the mass *m* (Table 1) of lithium hydroxide monohydrate (Thermo Scientific, 98%), sodium hydroxide (Fisher Thermo, 98.9%), or lithium perchlorate anhydrous (Thermo Scientific, 95%) to a 100 mL volumetric flask, filling the volumetric flask to its mark with water, and mixing liquid well until all solid was dissolved.

**Table 1:** Preparation of Aqueous Electrolytes

electrolyte	*x*	*m*_LiOH_ (g)	*m*_NaOH_ (g)	*m*_LiClO4_ (g)
8.0 M [LiOH]_*x*_–[NaOH]_(1–*x*)_	1.00	19.1		
	0.75	14.3	8.0	
	0.50	9.6	16.0	
	0.25	4.8	24.0	
	0.00		32.0	
6.0 M [LiOH]_*x*_–[NaOH]_(1–*x*)_	1.00	14.3		
	0.75	10.8	6.0	
	0.50	7.2	12.0	
	0.25	3.6	18.0	
	0.00		24.0	
6.0 M [LiOH]_*x*_–[LiClO_4_]_(1–*x*)_	1.00	14.3		
	0.75	10.8		16.0
	0.50	7.2		31.9
	0.25	3.6		47.9
	0.00			63.8

A Mettler Toledo SevenExcellence pH/Ion/C/DO meter S975–K with a InLab Expert Pro-ISM pH probe was used to measure the pH values of aqueous 6.0 M LiClO_4_ electrolyte. The pH values of the high-molarity aqueous NaOH and LiOH electrolytes used here cannot be measured because of alkaline error. Therefore, we were unable to measure the pH values of mixed-salt electrolytes. The pH values of aqueous 6.0 or 8.0 M LiOH and 8.0 M NaOH electrolytes were determined assuming complete salt dissociation, following reported work.^3^

Electrolytes were stored in plastic containers. PFOS solutions with 0.5 mM concentration were prepared by placing 10.76 mg of potassium perfluorooctanesulfonate (Synquest Laboratories, 95%) in a 50 mL Falcon tube and adding 40 mL of aqueous electrolyte. The PFOS-containing solution was subjected to ultrasonic agitation (Jeken PS-40A digital ultrasonic bath, 240 W, 10 L) for 5 min followed by sitting undisturbed for 30 min, to ensure proper mixing and dissolution. Chemicals were weighed on a Sartorius A 120 S analytical or a Mettler Toledo AT201 analytical balance. A Mettler Toledo SevenExcellence pH/Ion/C/DO meter S975–K with a InLab Expert Pro-ISM pH probe was used to measure the pH value of the aqueous LiClO_4_ electrolyte. The pH values of aqueous 6.0 M NaOH and LiOH electrolytes were obtained by calculations, as detailed in ref (3). A fluoride ion selective electrode (Thermo Scientific Orion, 9609BNWP) together with a total ionic strength adjustment buffer solution was used to quantify fluoride ions in aqueous solutions, as described in ref (3).

### 4.3 Physical Characterization

XPS data were obtained using a Kratos Axis Ultra XPS instrument with a monochromatized Al Kα source. Samples were washed with water and dried before affixing them to double-sided adhesive copper tape. The instrument operated at 200 W and 15 kV with a base pressure of 3.0 × 10^–8^ mbar. Survey scans covered 0–1200 eV with a step size of 1 eV, dwell time of 200 ms, and analyzer pass energy of 140 eV, averaged over 5 scans. High-resolution core level scans followed with a 0.1 eV step size, 260 ms dwell time, and 20 eV pass energy, averaged over 5 scans. Binding energies were calibrated against the C 1s peak at 284.8 eV.^28^ Data analysis using CasaXPS (Version 2.3.24) to obtain binding energies and quantitative peak areas included Shirley background subtraction,^64^ Gaussian/Lorentzian envelope peak fitting, and quantification using instrument-specific atomic sensitivity factors derived from standard materials. Peak area quantifications gave surface elemental content relative to total surface carbon content, with an 8% relative error, derived from the standard deviation of triplicate measurements.

SEM imaging of [NiFe]–(OH)_2_–hydrophilic carbon fiber paper anodes pre and post electrocatalysis were collected at UR-Nano, using a Zeiss Auriga scanning electron microscope, equipped with a Schottky field emission emitter, and operated at 20.00 kV with a working distance of 5.1 mm. EDX spectroscopy was performed on electrodes using a SEM-integrated EDAX Octane elect plus with silicon drift detector (SDD) spectrometer.

XRD data were collected at room temperature (298 K) using a Rigaku XtaLAB Synergy-S diffraction system equipped with a HyPix-6000HE HPC detector. Powder samples were affixed to a Nylon loop (0.1 mm ID) with a light coating of viscous oil. CuKα radiation (λ = 1.54184 Å) was generated by a PhotonJet-S microfocus source at 50 kV, 1 mA. Two combination ω–φ “Gandolfi” scans were performed, each for 300 s: (1) ω from −62.00° to 31.00° and φ rotated through 720°, at θ = −42.127 and κ = 70.000°; (2) ω from −31.00 to 61.00 degrees and φ rotated through 720 degrees, at θ = 40.877 and κ = −70.00°. The sample-to-detector distance was 34 mm.

### 4.4 Two-Dimensional (^7^Li or ^23^Na)–^19^F-HOESY-NMR

Two-dimensional NMR data were acquired on a 500 MHz JEOL NMR spectrometer. Each sample contained 750 μL of the respective electrolyte. All NMR experiments were performed at 25 °C. The collected spectra were analyzed using Delta version 6.2.0 and processed in Igor version 8.04. The 2D ^7^Li–^19^F-HOESY-NMR experiments were performed to measure the ^7^Li–^19^F NOE correlations. The fluorine axis sweep width was set to 5885 Hz and with 375 increments giving a resolution of 15.7 Hz. The lithium axis sweep width was set to 1943 Hz with 100 increments giving a resolution of 19.4 Hz. A total of 64 scans were needed to identify the ion interactions, which resulted in a data acquisition time of 7.5 h per sample. Likewise, 2D ^23^Na–^19^F-HOESY-NMR data were collected to measure the ^23^Na–^19^F NOE correlations. The fluorine axis sweep width was set to 5885 Hz and with 410 increments giving a resolution of 15.7 Hz. The sodium axis sweep width was set to 1323 Hz with 256 increments giving a resolution of 13.2 Hz. A total of 64 scans were used, which resulted in a data acquisition time of 7.5 h per sample.

### 4.5 Boiling Point Determination

The boiling point of 8.0 M aqueous LiOH was determined using a Thiele tube (150 mm length, 25 mm diameter, Eisco), which was securely clamped to a ring stand and filled with clear mineral oil (heavy, Fisher Chemical) up to a level of 1 cm above the apex of the top triangular arm of the Thiele apparatus. A thermometer (−10 to 260 °C, Durac Plus, SP Bel-Art) was carefully inserted into a one-holed rubber stopper that fit the Thiele tube and that had a slit down one side. A Durham tube (6 mm × 30 mm, JRLGD) was affixed to the side of the thermometer using a small rubber band so that bottom of the Durham tube was at the same height as the bottom of the thermometer. One mL of aqueous 8.0 M LiOH solution was filled into the Durham tube, and a capillary tube (Drummond Scientific) was placed into the LiOH solution. The rubber stopper and thermometer assembly were positioned in the Thiele tube such that the aqueous LiOH sample was positioned centered in height inside the Thiele tube. The apparatus was gently heated using a Bunsen burner (H-5890, Humboldt) with a flame height maintained at approximately 4 cm, utilizing a back-and-forth motion along the arm of the Thiele tube. The heating rate was maintained at 10 °C min^–1^ until the temperature reached 90 °C. Subsequently, the heating rate was reduced to 5 °C min^–1^ until the observation of a continuous stream of bubbles from the capillary tube within the aqueous 8.0 M LiOH solution, at which point the Bunsen burner was removed. The Thiele tube was allowed to cool at room temperature, and the temperature was recorded when the bubble at the bottom of the capillary disappeared, indicative of the boiling point.^65^

## Supporting Information Available

Catalyst and electrolyte characterization data (PDF)

## Abbreviations

CFP: carbon fiber paper
*E*_app_: applied potential
EDL: electrochemical double layer
EDX: energy-dispersive X-ray spectroscopy
HOESY: heteronuclear Overhauser enhancement spectroscopy
NMR: nuclear magnetic resonance
NOE: nuclear Overhauser effect
OCP: open circuit potential
PFAS: per- and polyfluoroalkyl substances
PFOS: perfluorooctane sulfonate
RHE: reversible hydrogen electrode
SEM: scanning electron microscopy
TISAB: total ionic strength adjustment buffer
UV: ultraviolet
XPS: X-ray photoelectron spectroscopy
XRD: X-ray diffraction
