# Supplementary material for: Role of LiOH in Aqueous Electrocatalytic Defluorination of Perfluorooctanoic Sulfonate: Efficient Li–F Ion Pairing Prevents Anode Fouling by Produced Fluoride
Source: ACS Catal. 2024 Oct 25;14(22):16577–88. doi: 10.1021/acscatal.4c04523 (PMC11574766; doi:10.1021/acscatal.4c04523)
Supplement: Supplementary file 1 — cs4c04523_si_001.pdf [file cs4c04523_si_001.pdf]

Supporting Information

Role of LiOH in Aqueous Electrocatalytic Defluorination of Perfluorooctanoic Sulfonate: Efficient Li–F Ion Pairing Prevents Anode Fouling by Produced Fluoride

Ziyi Meng,[†,#] *Madeleine K. Wilsey,*[†,#] *Astrid M. Müller**[†,‡,§]

[†]Materials Science Program, University of Rochester, Rochester, New York 14627, United States.

[‡]Department of Chemical Engineering, University of Rochester, Rochester, New York 14627, United States.

[§]Department of Chemistry, University of Rochester, Rochester, New York 14627, United States.

[#]Z.M. and M.K.W. contributed equally to this work.

*Email: astrid.mueller@rochester.edu

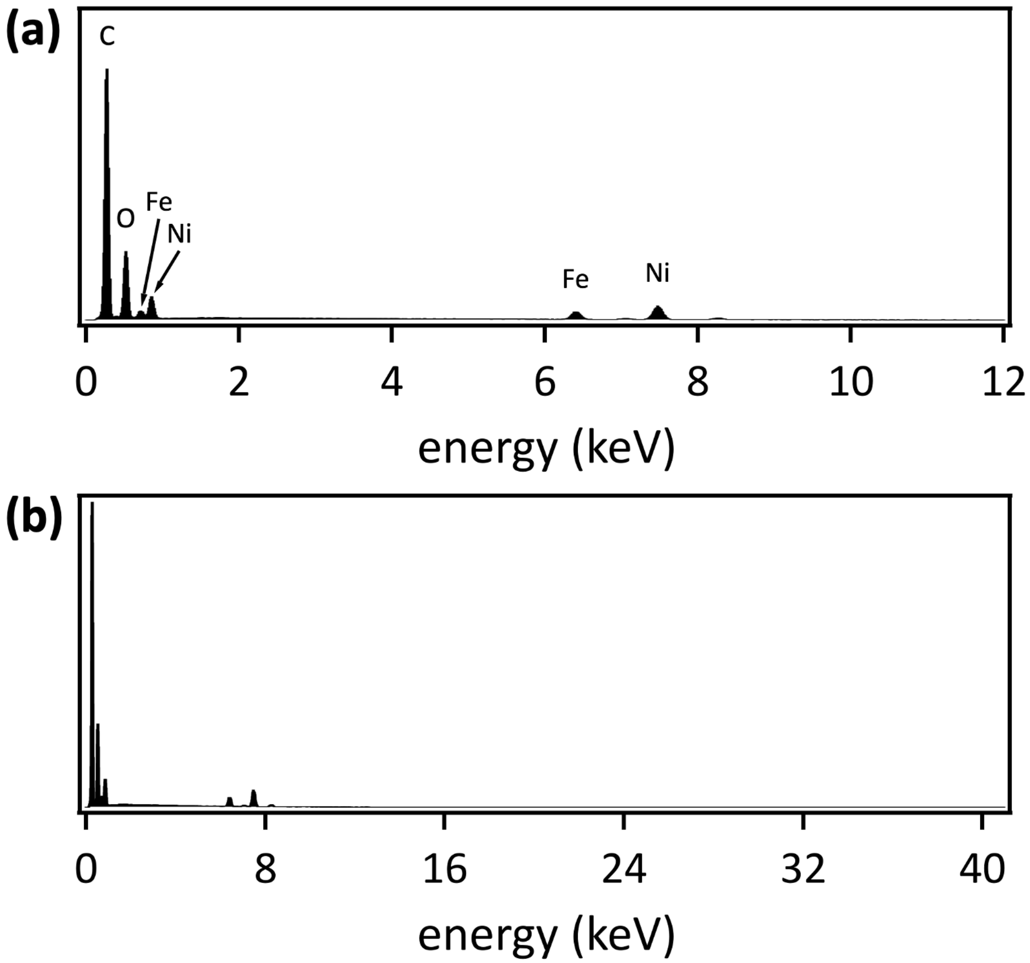

Figure S1. EDX spectra of laser-made $[Ni_{0.75}Fe_{0.25}]$-$(OH)_2$ nanosheets on hydrophilic carbon fiber paper in the (a) 0–12 and (b) 0–41 keV energy range.

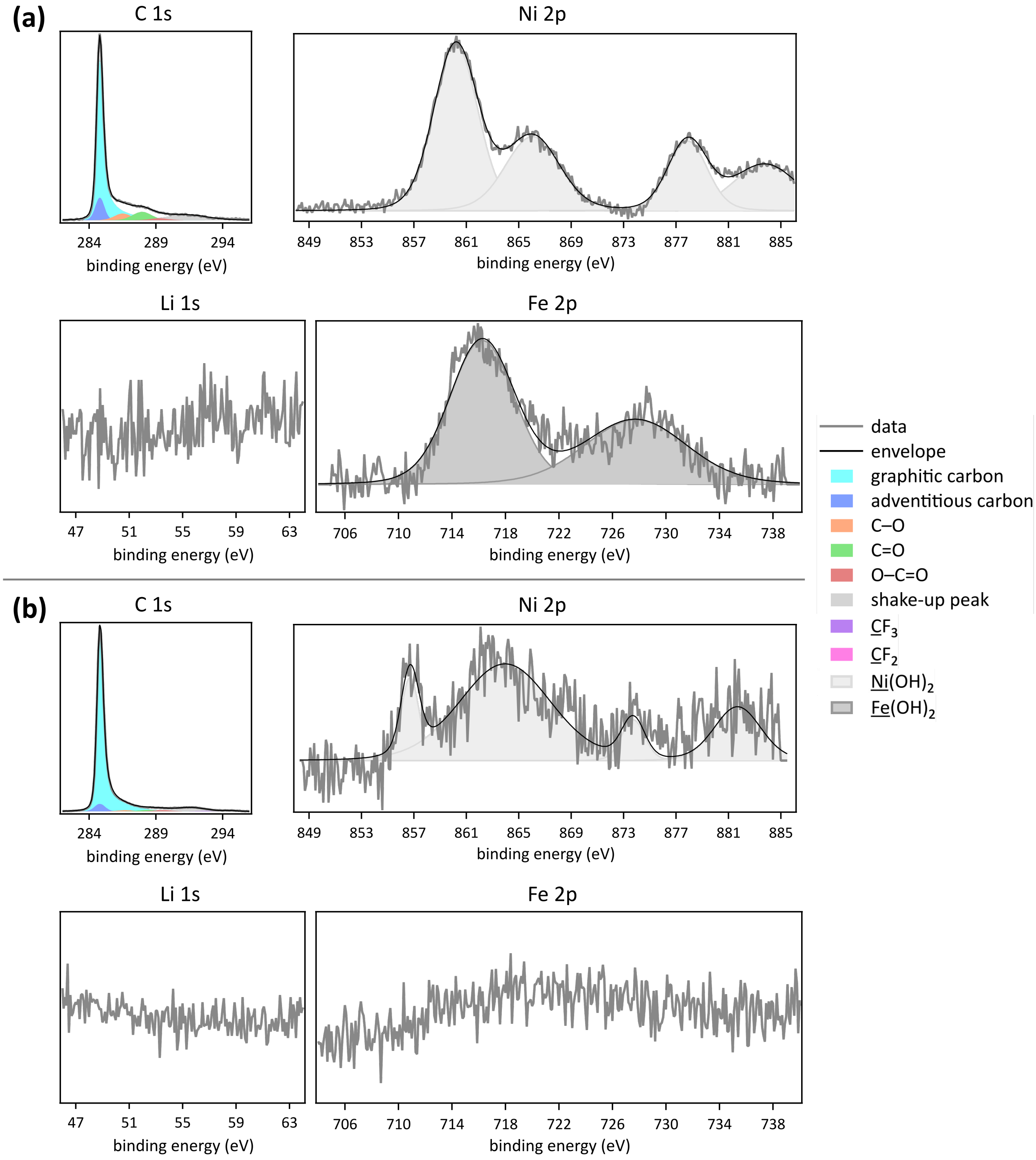

Figure S2. XPS data (a) pre electrocatalysis and (b) after PFOS defluorination (pulsed electrolysis for 60 cycles (1 cycle = 1 min at 1.6 V vs RHE, followed by 5 min at open circuit potential), deep UV light irradiation) in stagnant 8.0 M aqueous LiOH.

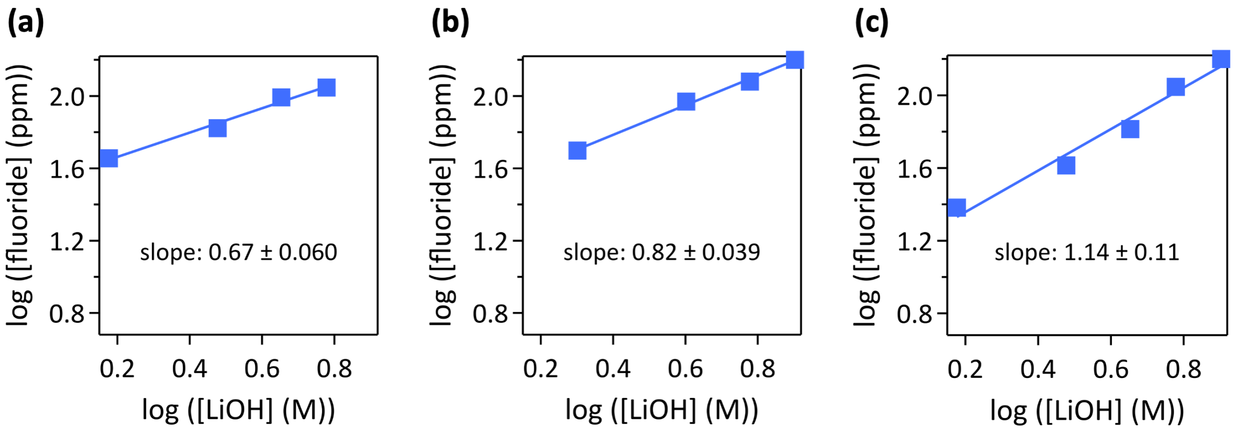

Figure S3. Log–log plots of PFOS defluorination (pulsed electrolysis for 60 cycles (1 cycle = 1 min at 1.6 V vs RHE, followed by 5 min at open circuit potential), deep UV light irradiation) in stagnant (a) 6.0 M or (b) 8.0 M aqueous $[LiOH]_x$–$[NaOH]_{(1-x)}$ or (c) 6.0 M aqueous $[LiOH]_x$–$[LiClO_4]_{(1-x)}$ (x = 1, 0.25, 0.5, 0.75, 0) electrolytes. The lines are linear fits.

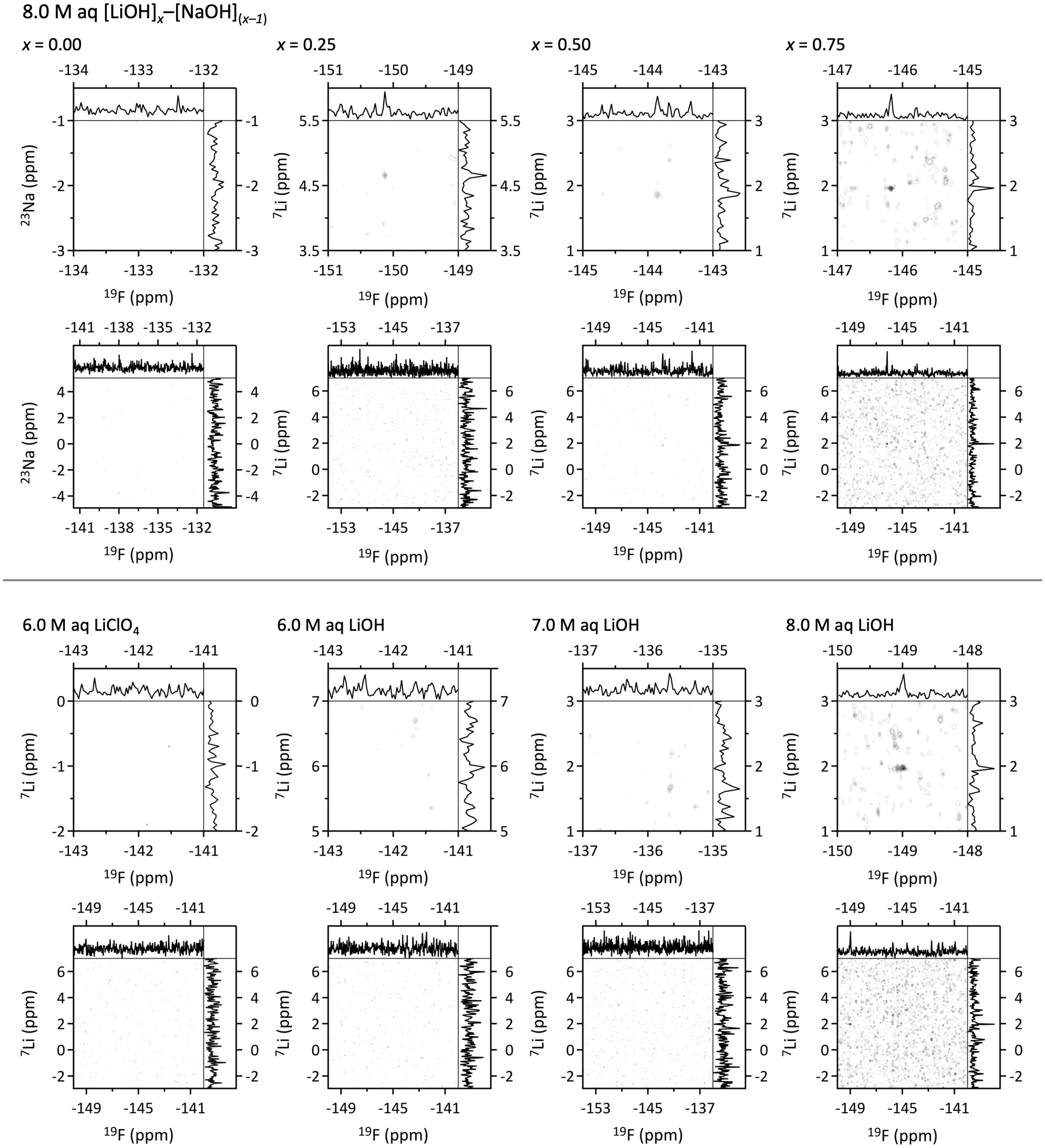

Figure S4. 2D-NMR data of PFOS defluorination (pulsed electrolysis for 60 cycles (1 cycle = 1 min at 1.6 V vs RHE, followed by 5 min at open circuit potential), deep UV light irradiation) in stagnant aqueous (aq) electrolytes.

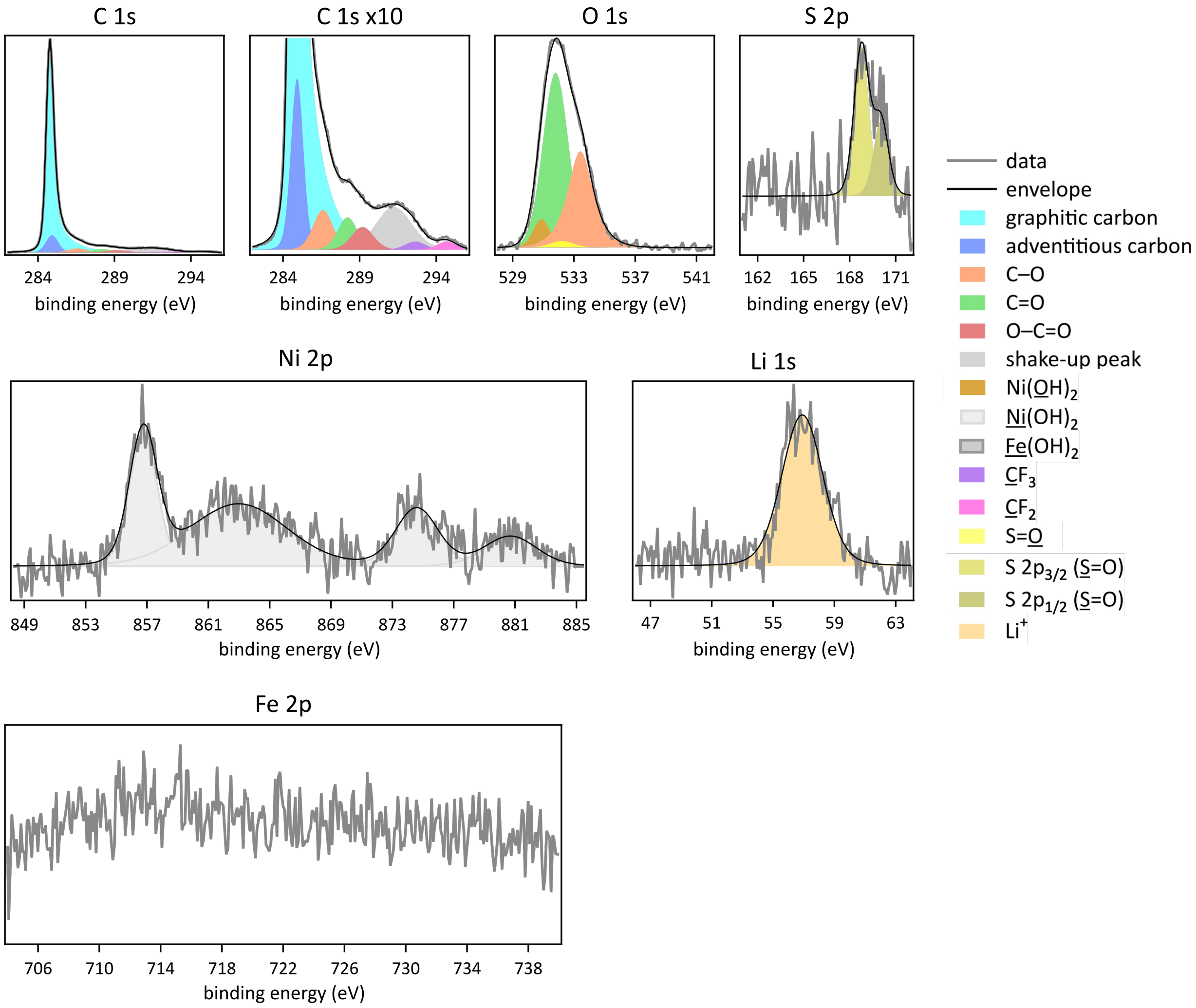

Figure S5. XPS data after PFOS defluorination (pulsed electrolysis for 60 cycles (1 cycle = 1 min at 1.6 V vs RHE, followed by 5 min at open circuit potential), deep UV light irradiation) in stagnant 6.0 M aqueous LiOH electrolyte.

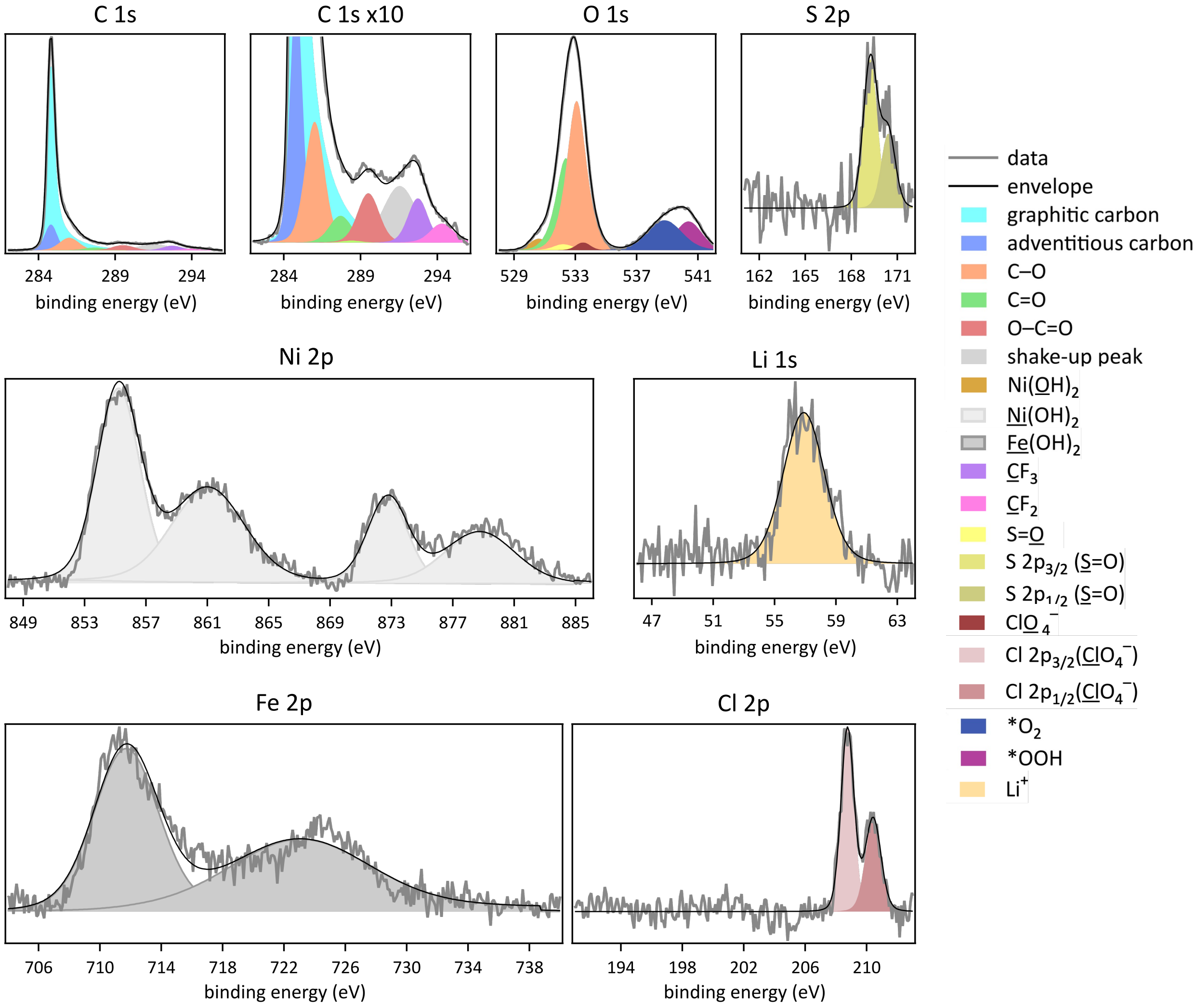

Figure S6. XPS data after PFOS defluorination (pulsed electrolysis for 60 cycles (1 cycle = 1 min at 1.6 V vs RHE, followed by 5 min at open circuit potential), deep UV light irradiation) in stagnant 6.0 M aqueous $LiClO_4$ electrolyte.
